# Overlap between telangiectasia and photoreceptor loss increases with progression of macular telangiectasia type 2

**DOI:** 10.1371/journal.pone.0224393

**Published:** 2019-10-28

**Authors:** Paul S. Micevych, Hee Eun Lee, Amani A. Fawzi

**Affiliations:** Department of Ophthalmology, Feinberg School of Medicine, Northwestern University, Chicago, Illinois, United States of America; Massachusetts Eye & Ear Infirmary, Harvard Medical School, UNITED STATES

## Abstract

**Objectives:**

To examine the topographical correlation between ellipsoid zone loss and telangiectasia in the deep capillary plexus in patients with macular telangiectasia type 2 (MacTel).

**Methods:**

38 eyes (20 subjects) diagnosed with MacTel were imaged with OCTA between March 2016 and June 2019 in this single center, cross-sectional observational study. The *en face* OCTA and OCT were evaluated for areas of deep capillary plexus telangiectasia and ellipsoid zone loss, respectively, and their outlines were superimposed to study their overlap (mm^2^). The primary outcome was percentage of overlap and its relationship to MacTel stage. Secondary outcomes included the relationship between neovascularization and hyperreflective foci as well as correlations between ellipsoid zone loss, deep capillary plexus telangiectasia and visual acuity.

**Results:**

In nonproliferative MacTel stage, ellipsoid zone loss was localized to margins of telangiectatic areas (mean overlap = 15.2%). In proliferative stages, ellipsoid zone loss showed a higher degree of overlap with telangiectatic areas (mean overlap = 62.8%). Overlap increased with advancing MacTel stages, with an overall average of 45.3%. Overlap correlated highly with ellipsoid zone loss (*r* = 0.831; *p*<0.0001). Telangiectasia was present in all 38 eyes (range: 0.08mm2–0.99mm^2^), while ellipsoid zone loss was absent in 6 (range: 0.00–3.32mm^2^). Visual acuity correlated most strongly with ellipsoid zone loss (*r* = 0.569; *p* = 0.0002), followed by overlap (*r* = 0.544; *p* = 0.0004), and finally, telangiectasia (*r* = 0.404; *p*<0.0118). Presence of hyperreflective foci on OCT correlated with the presence and intraretinal location of neovascularization.

**Conclusions:**

Ellipsoid zone loss occurs at the margins of deep capillary plexus telangiectasia in nonproliferative MacTel, with progressively increasing overlap as MacTel advances, peaking in proliferative disease. Deep capillary plexus telangiectasia and its overlap with ellipsoid zone loss are two promising markers of nonproliferative MacTel, while hyper-reflective foci are markers for proliferative MacTel.

## Introduction

Idiopathic macular telangiectasia type 2 (MacTel) is a rare, acquired macular disease that leads to progressive vision impairment. The pathophysiology of MacTel is believed to be neurodegenerative, characterized by primary loss of supportive Müller cells with secondary, reactive vascular changes.[1] Characteristic vascular and neurodegenerative findings define the disease; these include loss of foveal translucency, crystalline deposits, intra-retinal cavitary lesions, loss of macular pigment, widened capillary spaces, right-angle venules, capillary ectasia, and late-stage neovascularization (outer retinal progressing to subretinal).[2–6] In recent years, optical coherence tomography angiography (OCTA) has been used to better characterize the macular capillary network in MacTel. OCTA has been shown superior to fundus fluorescein angiography (FFA) in visualizing the deep capillary plexus (DCP).[7] The DCP is of particular interest in MacTel as it harbors the majority of vascular changes[3], is poorly perfused (as marked by lower parafoveal vascular density[8, 9]), and may show evidence of MacTel before the superficial capillary plexus (SCP).[10]

MacTel is classically viewed as having nonproliferative and proliferative stages based on the absence or presence of neovascularization.[11] Nonproliferative MacTel has been further subclassified based on the topographical location of vascular anomalies in the SCP and DCP on OCTA.[8, 12] Based on depth of neovascularization, proliferative MacTel can be further subclassified[13] based on location of the neovascularization in the outer retina or in the subretinal space.[14, 15] The classification of MacTel is evolving, and a staging system using OCT and autofluorescence is on the horizon. The newly proposed classification system by Chew et al. delineates MacTel into 7 stages (0–6) based on the presence and location of ellipsoid zone (EZ) loss, presence and location of macular pigmentary deposits, presence of OCT hyperreflective foci (HF), and the presence of subretinal neovascularization–factors shown most influential in progression of disease[16]. One of the limitations of this recent classification is that it does not consider the vascular findings of MacTel, since OCTA was not included in these baseline studies. Given our goal of characterizing the vascular telangiectasia, we relied mainly on OCTA MacTel classifications, with slight modification.[8, 12]

The pathogenesis of the vascular abnormalities in MacTel is thought to be intricately related to neurodegeneration. Studies in mouse models have shown that selective ablation of Müller cells leads to photoreceptor apoptosis and outer retinal neovascularization.[17] These studies suggest a possible causal link between neurodegenerative loss of the photoreceptors and subsequent angiogenesis in the proliferative phase of MacTel, though this relationship has not been explored in human MacTel. EZ loss, a marker of the zone of photoreceptor degeneration, has been shown to be an independent marker of disease severity[18–20] and visual function[21, 22] in MacTel patients. Using OCTA in proliferative MacTel, researchers have shown that the neovascular lesion and the zone of EZ loss show almost complete overlap in eyes with outer retinal neovascularization.[13, 21] However, to better define the entire spectrum of MacTel pathology, it is important to explore these neurovascular pathologies in eyes that span the entire disease spectrum, especially those in the earlier stages.

The relationship between EZ loss and the nonproliferative vascular abnormalities in MacTel has not been previously explored. Furthermore, while EZ loss is a critical component of the pathology, there are no defined markers for the early vascular changes in MacTel. In this study, we therefore set out to study the topographic overlap between DCP telangiectasia (a marker of early vascular pathology in MacTel) and the zone of EZ loss (a marker of neurodegenerative pathology). Quantifiable markers of early MacTel prior to development of EZ loss are scarce, so we explored DCP telangiectasia and the overlap between it and the EZ loss as two potential novel parameters. Based on the strong evidence for neurovascular basis of the pathology in MacTel, we hypothesized that DCP telangiectasia would topographically overlap with regions of EZ loss.

## Methods

### Subjects

This was an observational, cross-sectional study of 20 patients (38 eyes) diagnosed with MacTel between March 2016 and June 2019 in the Department of Ophthalmology at Northwestern University in Chicago, Illinois. One subject underwent follow up OCTA at 6 months. Written informed consent was obtained from all participants. This research was approved by the Institutional Review Board of Northwestern University, followed the tenets of the Declaration of Helsinki, and was performed in accordance with the Health Insurance Portability and Accountability Act regulations.

Patients underwent OCTA imaging of both eyes during a single clinic visit, as well as color fundus photography, fundus autofluorescence (FAF), and FFA as clinically indicated. Diagnosis of MacTel was made based on characteristic funduscopic findings (perifoveal translucency, inner retinal crystals, non-tapering angled venules, and ectatic capillaries) with typical FFA patterns (telangiectatic capillaries with late-phase hyperfluorescence). All subjects underwent a complete ophthalmological examination, including corrected Snellen visual acuity, which in this study is expressed using the LogMAR scale.

Subjects were staged based on OCTA criteria as follows. Stage I is defined by vascular anomalies temporal to the fovea in the deep or superficial capillary plexus. Stage II is defined by multi-quadrant vascular anomalies in the deep or superficial capillary plexus. Stage III is defined by proliferative MacTel, which we further subdivide into stage IIIa with outer retinal neovascularization and stage IIIb with subretinal neovascularization. For future reference, subjects were also staged using the recently proposed Chew et al. classification.[16] This proposed classification is described in **S1 Appendix** and its staging criteria are listed in **S2 Appendix.**

### OCTA protocol

All subjects were imaged with RTVue-XR Avanti system (Optovue Inc., Fremont, California, USA). 3x3 mm OCTA scans were acquired for this study. The system captures two consecutive B-scans at each location, including 304 A-scans at a rate of 70,000 scans per second. RTVue-XR Avanti uses a bandwidth of 45mm and a light source centered on 840nm. RTVue-XR Avanti utilizes split-spectrum amplitude-correlation angiography (SSADA) software (version 2017.1.0.151). The SSADA algorithm generates angiographic flow information by quantifying the difference in OCT amplitude (decorrelation) between two consecutive B-scans. Superficial projection artifacts were reduced by using the built-in flow artifact removal software.

### Image analysis

Default segmentation parameters were used to study the macular capillary beds separately as *en face* angiograms of the SCP and DCP. The SCP is segmented from the inner limiting membrane (ILM) to 55μm above the inner plexiform layer (IPL). The DCP is segmented from 6μm above the IPL to 50μm below the IPL. *En face* OCT of the EZ was segmented by manually adjusting the choriocapillaris slab to center onto the EZ, as visualized on cross-sectional OCT. We imported 3x3mm images of the SCP, DCP, and EZ into Photoshop software (Adobe systems, San José, CA) for superimposition and area calculations.

Telangiectatic vessels in the DCP were identified as non-tapering or dilated capillaries and traced individually. One or more circumscribed areas encompassing the telangiectatic vessels were subsequently quantified using Photoshop. The primary exclusion criteria for telangiectatic-appearing changes was superficial vessel artifact, so all focal areas suggestive of DCP telangiectasia were cross-referenced for overlying SCP artifact. A second masked examiner evaluated telangiectasia in a random sample of 10 eyes to study reliability of this measurement. Areas of EZ loss were identified on *en-face* OCT as dark or hyporeflective regions that demonstrated a concurrent EZ defect on cross-sectional OCT, as has been done in prior studies.[13] Areas of *en-face* hyporeflectivity without a topographically associated EZ defect on cross-sectional OCT were excluded due to minimize any potential for shadowing from overlying retinal pathology. Subsequently, maps of DCP telangiectasia were superimposed onto EZ loss maps to illustrate regional overlap. Areas of telangiectasia, EZ loss, and their overlap were quantified in mm^2^ on Photoshop. Total pixels were divided by the square resolution of each image (pixels/mm)^2^ to generate units of area in mm^2^. Percentage of overlap was calculated relative to telangiectasia and stratified by disease stage.

### Statistical analysis

Pearson correlation coefficients were calculated for relationships between area of EZ loss, telangiectasia, percent overlap, and visual acuity (LogMAR). As a proxy of inter-rater reliability, an intraclass correlation (ICC) value was calculated for telangiectasia area measurements comparing the two graders. Two-tailed *t-*tests were performed to evaluate the difference in percent overlap between groups of MacTel subjects using the OCTA and OCT-based stages. We performed all statistical analyses using JMP 14.0 software (SAS Institute, Cary, NC, USA) and significance was set at *p*<0.05.

## Results

**Fig 1:**
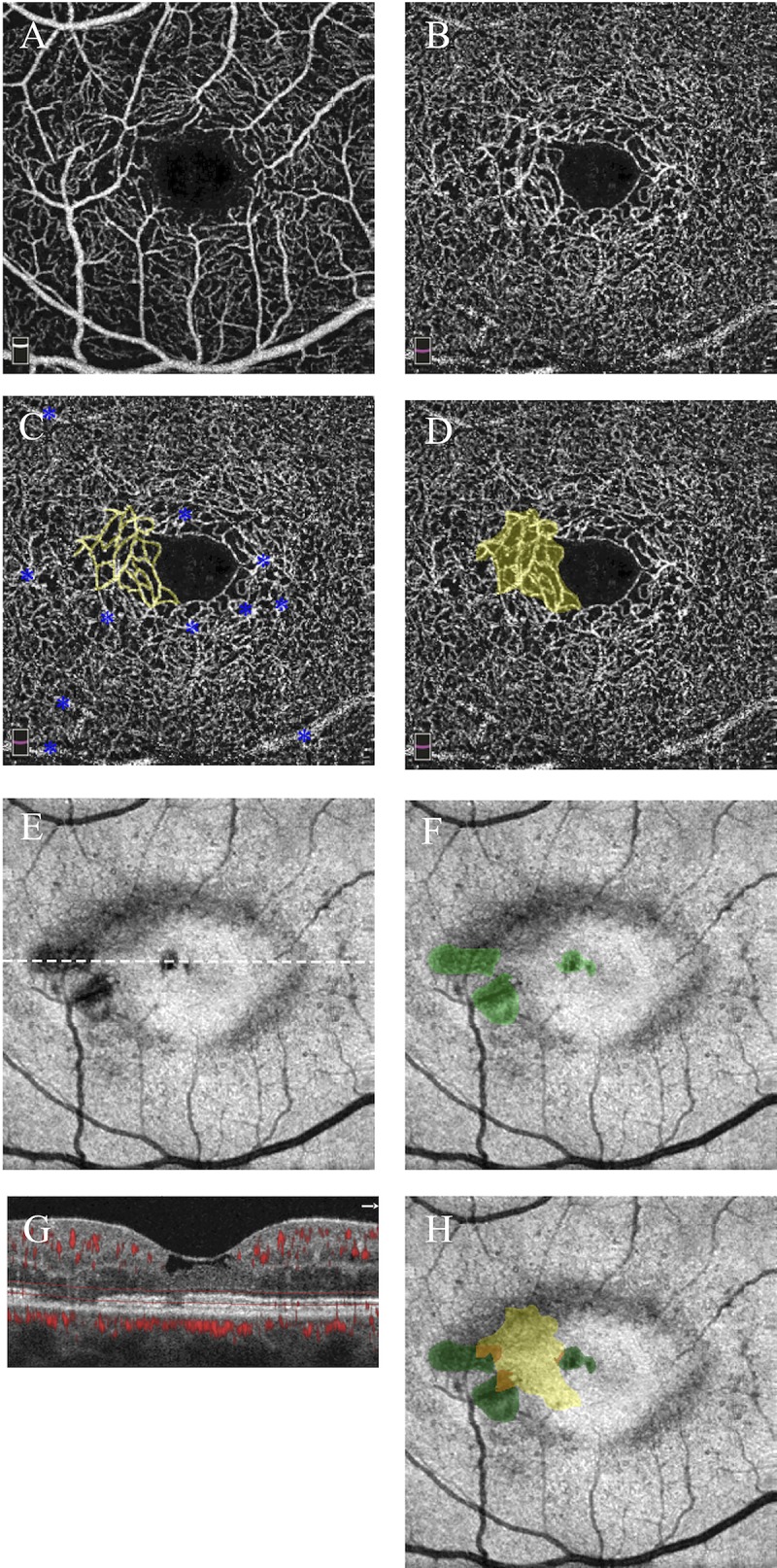
Minimal overlap between telangiectasia and photoreceptor loss in stage I (nonproliferative) MacTel. Right eye, 43-year-old woman. **A)** Superficial capillary plexus is shown for reference. **B)** Deep capillary plexus (DCP) shows the dilated temporal parafoveal capillary network. **C)** DCP is shown with the network of telangiectatic capillaries outlined in yellow; blue asterisks label artifact from superficial vessels. **D)** DCP is shown with the area of telangiectasia pseudocolored in yellow. **E**) E*n face* OCT image in plane with the ellipsoid zone (EZ) shows dark areas corresponding to photoreceptor loss; white dotted line corresponds to accompanying cross-sectional image. **F)** *En face* EZ map is shown with photoreceptor loss pseudocolored in green. **G)** Cross-sectional image is shown with segmentation lines (red) used to generate the *en face* EZ map. **H)** *En face* EZ map is shown with superimposed areas of telangiectasia (yellow), photoreceptor loss (green), and regions of overlap (orange), demonstrating minimal (10.2%) overlap.

**Fig 2:**
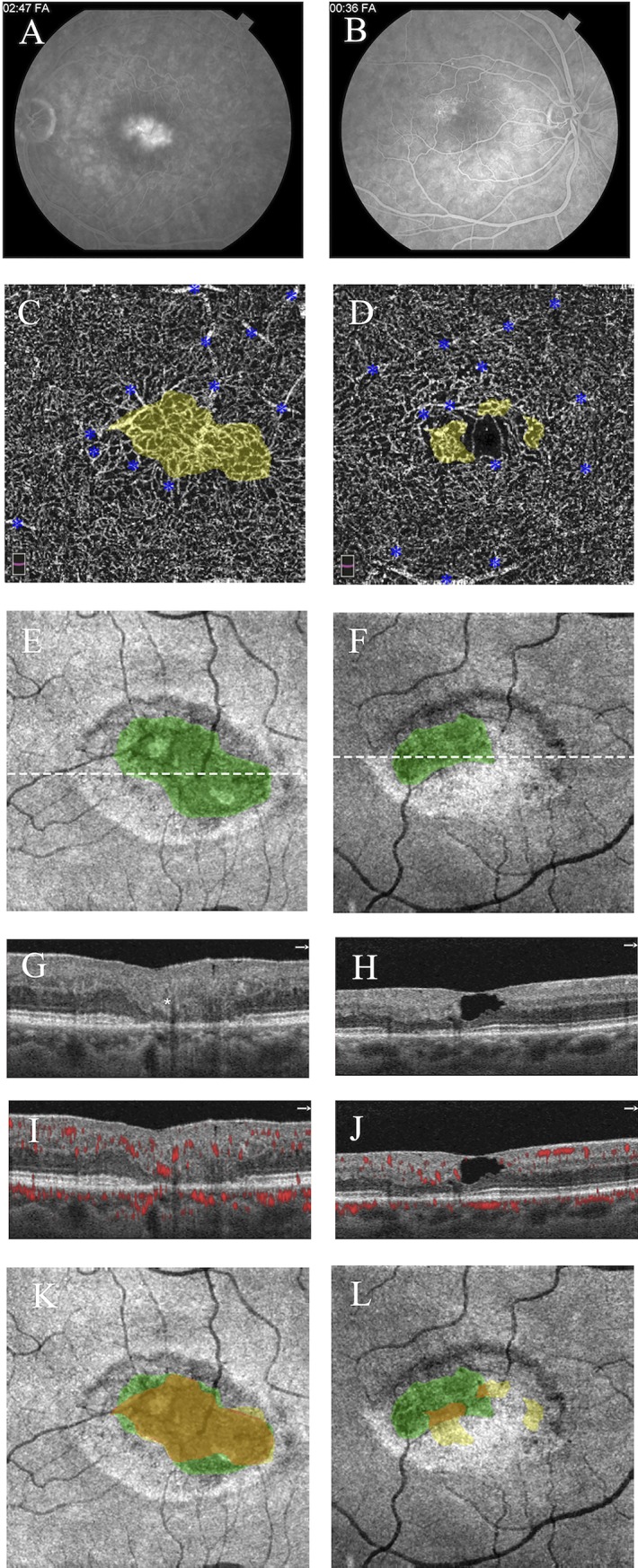
**Greater overlap between telangiectasia and photoreceptor loss in stage IIIa (outer retinal proliferative; left) than stage II (nonproliferative; right) MacTel.** Left eye (left column) and right eye (right column), 53-year-old man. **A,B)** Fluorescein angiograms are shown in late phase (left eye) and early-mid phase (right eye); parafoveal telangiectasias and leakage are identified bilaterally, greater in the left eye. **C,D)** Deep capillary plexuses with superimposed pseudocolored areas of telangiectasia (yellow) show invasion of foveal avascular zone, greater in the left eye; blue asterisks label artifact from superficial vessels. **E,F)** *En face* OCT images in the plane of the ellipsoid zone (EZ) show areas of pseudocolored photoreceptor loss (green), greater in the left eye; white dotted lines correspond to accompanying cross-sectional images. **G,H)** Cross-sectional images corresponding to the *en face* EZ maps show hyperreflective foci (marked by white asterisk) only in the left eye. **I,J)** Cross-sectional images with flow overlays corresponding to the *en face* EZ maps show outer retinal neovascularization only in the left eye. **K,L)** *En face* EZ maps are shown with superimposed areas of telangiectasia (yellow), ellipsoid loss (green), and overlap (orange), demonstrating greater overlap in the left eye (92.9%) than right eye (27.1%).

Subject demographics, visual acuity, and OCTA-based disease stages are reported in **Table 1**. For reference, recently proposed OCT-based (Chew et al.) disease stages are listed in **S2 Appendix**. To assess reliability of the telangiectasia measurement, we quantified inter-rater reliability (ICC = 0.85; *p* = 0.0002) in 10 randomly selected eyes. Superimposition of telangiectasia maps onto areas of EZ loss unveiled a dynamic relationship between DCP telangiectasia and EZ loss, as illustrated in **Figs 1 and 2**. All 38 eyes (100%) showed evidence of DCP telangiectasia on OCTA, whereas only 32 of 38 eyes (84.2%) demonstrated evidence of EZ loss. EZ loss was predominantly localized to margins of telangiectasia areas in early OCTA-based stages (stages I-II). In more advanced, proliferative disease (stages IIIa-IIIb), EZ loss overlapped more centrally with areas of telangiectasia and neovascularization.

**Table 1:** Study population demographics, clinical characteristics, and staging of MacTel.

Characteristic	Mean ± SD (range)
Subjects	20
Eyes	38
Age (years)	60.8 ± 9.8 (43–85)
Sex Male	
	15
Female	23
Laterality OD	19
OS	19
Visual Acuity LogMAR	
	0.27 ± 0.25 (-0.1–0.9)
Snellen	20/36 (20/16–20/160)
OCTA Stage I	
	7
II	7
IIIa	9
IIIb	15

SD = standard deviation

**Fig 3:**
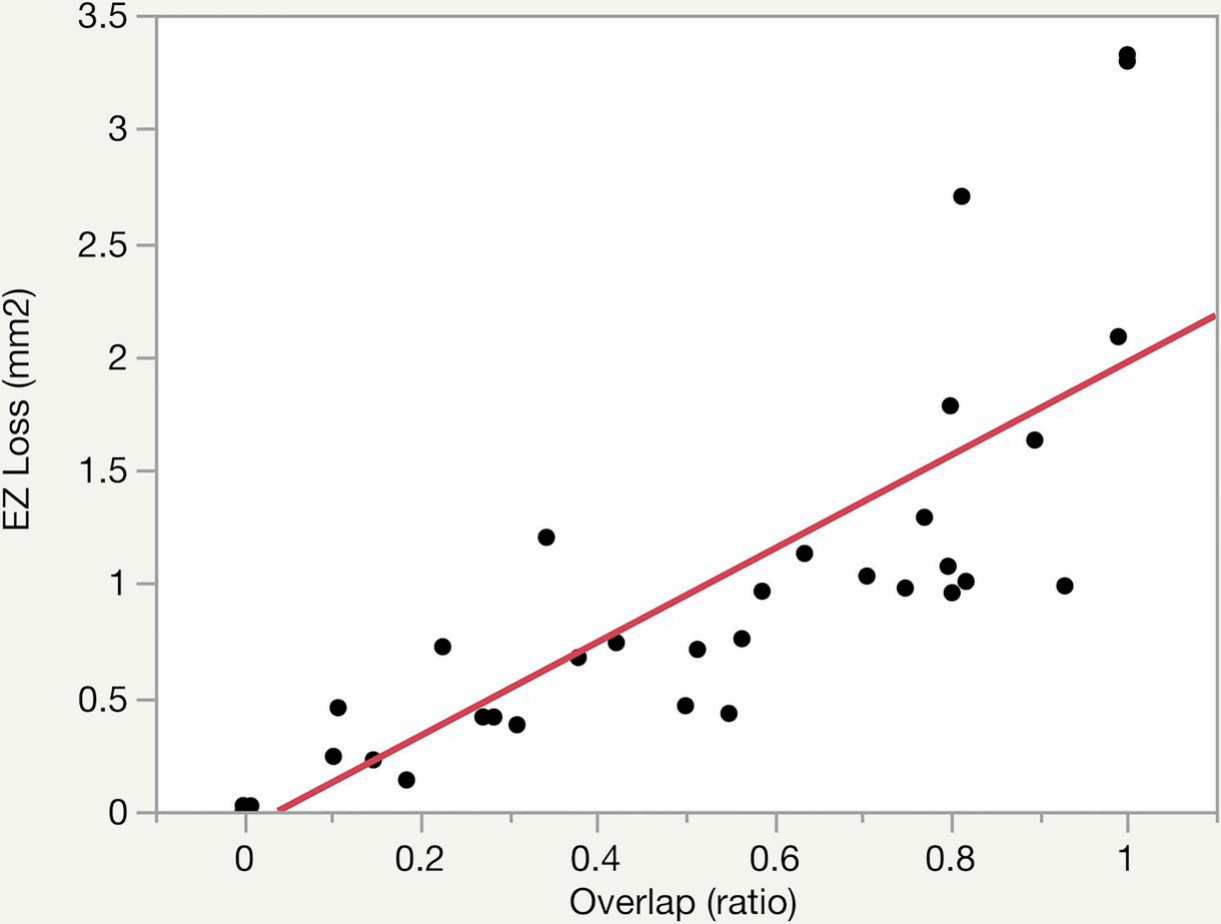
Relationship between photoreceptor loss (EZ loss) and its overlap with telangiectasia (overlap). A significant, positive linear correlation is illustrated between photoreceptor (ellipsoid zone; EZ) loss and overlap (*r* = 0.831; *p*<0.0001).

Overall, the mean percentage of telangiectasia-EZ overlap was 45.3% (standard deviation 0.35; range 0.0–1.0). As shown in **Fig 3**, the degree of overlap increased with progressive EZ loss, showing a strong linear correlation (*r* = 0.831; *p*<0.0001). Visual acuity correlated most strongly with EZ loss (*r* = 0.569; *p* = 0.0002), followed by overlap (*r* = 0.544; *p* = 0.0004), and finally, telangiectasia (*r* = 0.404; *p*<0.0118). Using OCTA staging, the overlap increased progressively with advancing disease stage; stage I: 2.22% stage II: 28.2%, stage IIIa: 47.4%, stage IIIb: 72.0%. There were statistically significant differences between nonproliferative stage I and nonproliferative stage II (p<0.0441), as well as between proliferative stages (IIIa-IIIb; 62.8%) and nonproliferative stages (I-II; 15.2%; *p*<0.0001), and between outer retinal and subretinal proliferative stages (IIIa and IIIb; *p*<0.0447). We also evaluated our findings using OCT-based Chew et al. staging[16], as shown in **S3 Appendix**. Similar to the OCTA staging mean overlap generally increased with advancing disease stage; stage 0: 0%, stage 1: 16.8%, stage 2: 13.9%, stage 3: 45.9%, stage 4: 51.2%, stage 5: no subjects, stage 6: 72.0%. There were significant differences between grouped stages 0–2 and 3–5 (*p* = 0.0003), as well as between grouped stages 3–5 and stage 6 (*p* = 0.0410).

**Fig 4:**
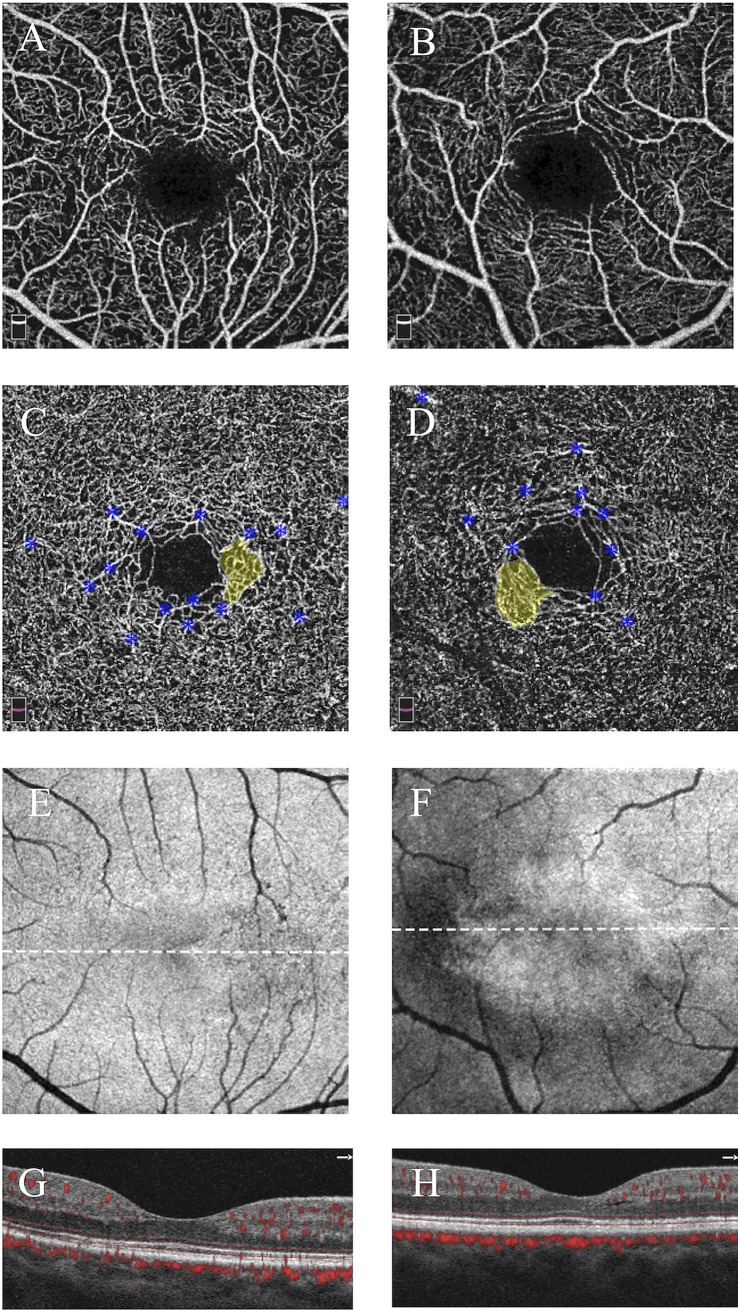
Two presentations of stage I (nonproliferative) MacTel showing telangiectasia without photoreceptor loss. Left eye, 48-year-old man (left column); right eye, 60-year-old woman (right column). **A,B)** Superficial capillary plexuses show largely normal architecture in both eyes. **C,D)** Deep capillary plexuses are shown with pseudocolored areas of parafoveal telangiectasia in yellow; blue asterisks label projection artifact from superficial vessels. **E,F)** *En face* OCT images in the plane of the ellipsoid zone show no areas of loss in either eye; white dotted lines correspond to accompanying cross-sectional images. **G,H)** Cross-sectional images with flow overlay show a continuous ellipsoid zone without areas of breakdown.

**Fig 5:**
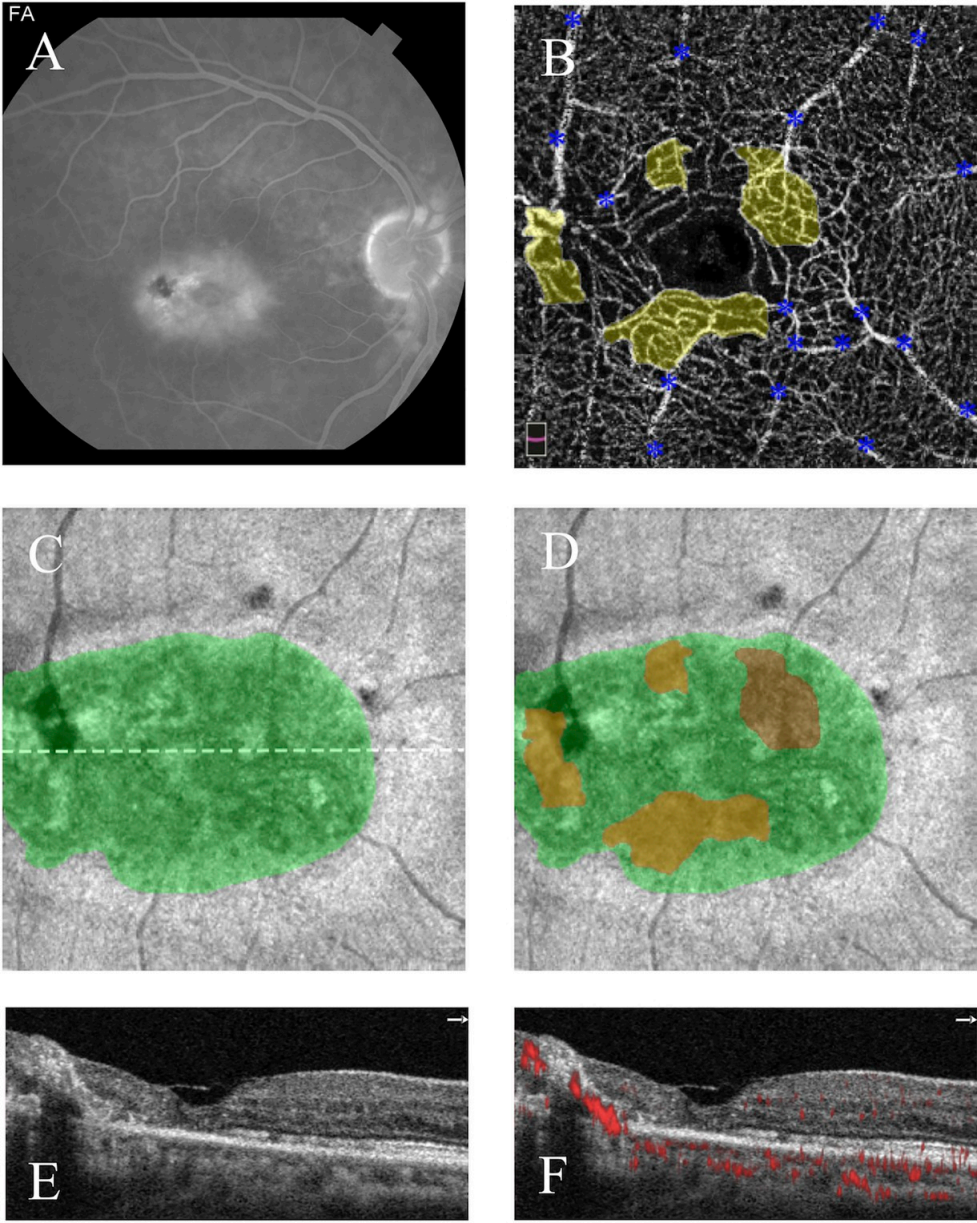
Maximal overlap between telangiectasia and photoreceptor loss in stage IIIb (subretinal proliferative) MacTel. Right eye, 79-year-old woman. **A)** Fundus fluorescein angiography (late phase) shows hyperfluorescence from subretinal neovascularization. **B)** Deep capillary plexus shows parafoveal telangiectasia (pseudocolored in yellow) and widened inter-capillary space; blue asterisks label projection artifact from superficial vessels. **C)** E*n face* OCT in the plane of the ellipsoid zone is shown with areas of photoreceptor loss pseudocolored in green; white dotted line corresponds to accompanying cross-sectional images. **D)** E*n face* OCT in the plane of the ellipsoid zone is shown with the superimposed pseudocolored map of ellipsoid loss (green) and overlap (orange), demonstrating maximal (100%) overlap. **E,F)** Cross-sectional images without and with flow overlays, respectively, show subretinal neovascularization and its association with hyperreflective foci (marked by the white asterisk).

Two eyes with DCP telangiectasia without EZ disruption are displayed in **Fig 4**. An eye with stage IIIb subretinal proliferative MacTel demonstrating 100% overlap between EZ loss and telangiectasia is shown in **Fig 5**. Only mild telangiectatic change is visible on fundus photography or FFA, but significant distortion and dilation is seen on OCTA of the DCP.

**Fig 6:**
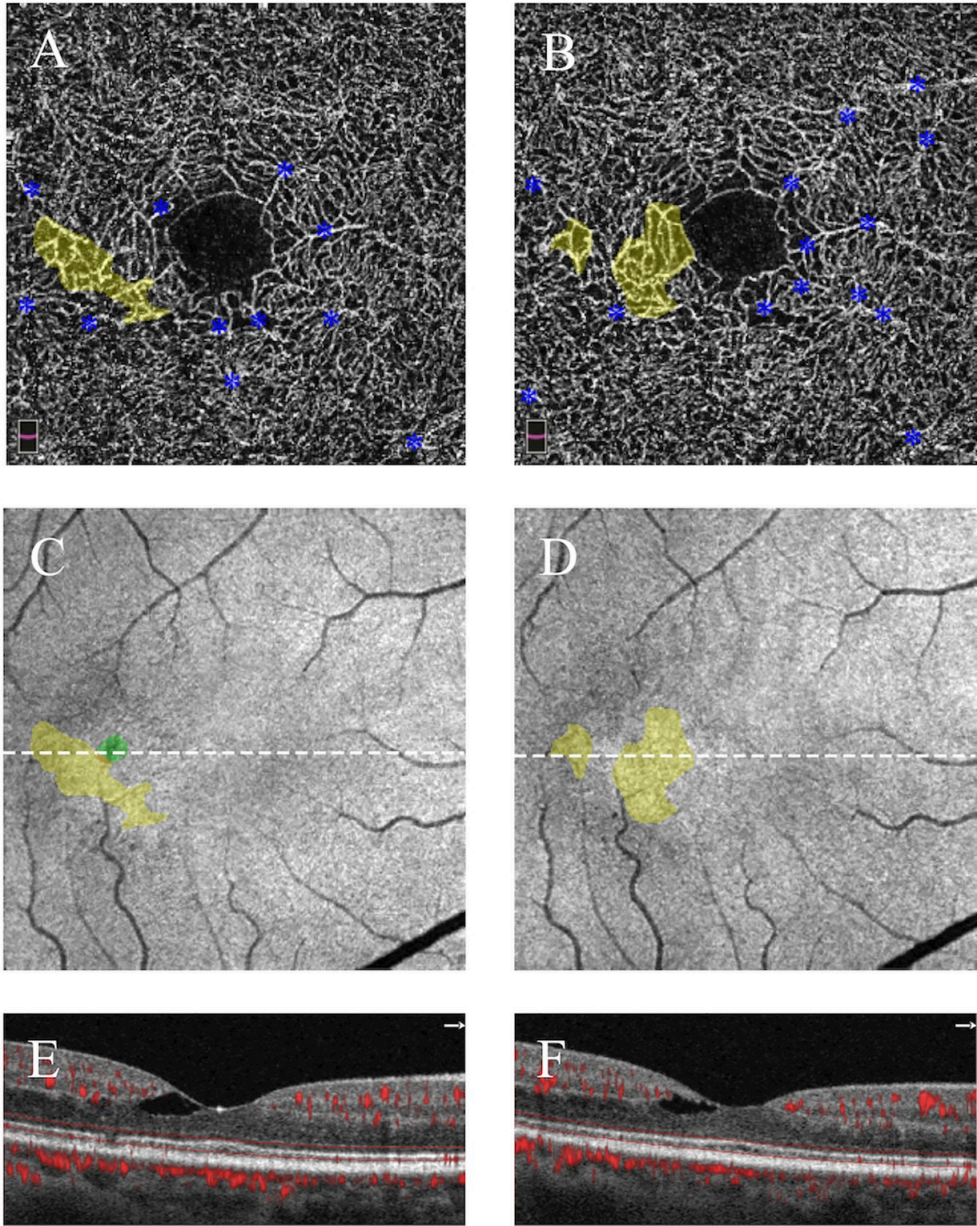
Dynamic changes in telangiectasia and ellipsoid zone in stage I (nonproliferative) MacTel. Right eye, 61-year-old woman; baseline imaging (left column), 6-month follow-up (right column). **A,B)** Deep capillary plexuses at baseline and follow-up show dynamic areas of telangiectasia (pseudocolored in yellow); blue asterisks mark projection artifact from superficial vessels. **C,D)** *En face* images of the ellipsoid zone are shown at baseline and follow-up with pseudocolored areas of telangiectasia (yellow) and photoreceptor loss (green); white dotted line corresponds to accompanying cross-sectional images. Photoreceptor loss seen at baseline is no longer present on follow-up imaging. **E,F)** Cross-sectional images with flow overlays are shown at baseline and follow-up, demonstrating photoreceptor recovery.

Baseline and 6-month follow-up imaging were analyzed in one subject, as illustrated in **Fig 6**. DCP telangiectasia and EZ loss showed subtle topographical dynamic changes. Most prominently, the small area of EZ loss at baseline showed recovery and disappeared at 6 months.

The presence of HF on OCT reflected the presence and depth of neovascularization (**Figs 2 and 5)**. HF were present in 100% of subjects (15/15) with subretinal neovascularization (stage IIIb), 77.8% of subjects (7/9) with outer retinal proliferation (stage IIIa), and only 7.1% of subjects (1/14) without neovascularization (stage I-II).

## Discussion

In this pilot study, we sought to examine the intricate relationship between vascular and neurodegenerative changes in MacTel by exploring the topographical relationship between EZ loss and DCP telangiectasia. We originally hypothesized that areas of EZ loss would directly overlie areas of telangiectasia, but instead found that the topographical relationship varies by disease stage, as visualized in **Fig 2.** Qualitatively, telangiectasia was located along the margins of EZ loss in early MacTel (stages I-II; **Fig 1**) and more centrally overlapping in late, proliferative disease (stages IIIa-IIIb; **Fig 5**). Quantitatively, this was reflected by a stepwise increase in overlap with increasing disease stage as well as by the linear correlation between overlap and EZ loss, as shown in **Fig 3**.

The topographical relationship, with central photoreceptor loss surrounded by telangiectasia, is consistent with the prevailing hypothesis on MacTel pathogenesis. Neurodegenerative changes in MacTel, characterized by the loss of Müller cells and ensuing loss of photoreceptors, are believed to precede and cause the vascular abnormalities.[1, 17] Novel therapies for MacTel target Müller cell dysfunction through ciliary neurotrophic factor (CNTF) therapy and have been shown to slow photoreceptor degeneration in preclinical models by activating gp130 receptor in Müller cells.[23] Surgical implantation of slow-release intravitreal CNTF has shown promise in slowing retinal degeneration in subjects with MacTel.[24] From a pathophysiologic perspective, mouse models have shown that photoreceptor loss can promote a local pro-angiogenic environment leading to outer retinal neovascularization.[25–27] In the normal retina, photoreceptors synthesize and release an endogenous inhibitor of vascular endothelial growth factor A (VEGF-A), known as soluble VEGF receptor-1 (sFLT-1).[28] This molecule is thought to play an important role in establishing and maintaining the normal avascularity of the outer retina.[28] Using immunostaining methods, sFLT-1 levels have been shown to decrease in eyes with age-related macular degeneration, which, like MacTel, can exhibit vascular invasion of the outer retina.[28] Other factors that inhibit outer retinal neovascularization include very low-density lipoprotein receptor (VLDLR) expression.[25] VLDLR knockout mice exhibit retinal neovascular changes similar to those seen in type 3 neovascularization.[25] Derangements in these anti-angiogenic signaling mechanisms may explain the topographical relationship between EZ loss and telangiectasia in early MacTel, as well as the outer retinal vascular invasion in proliferative stages of MacTel.

Interestingly, vascular changes are already present in MacTel prior to the development of outer retinal neovascularization, most prominently in the DCP as reflected in the OCTA grading paradigm.[8, 12] It is not well understood whether the loss of photoreceptors plays a role in influencing these earlier vascular changes, prior to development of outer retinal neovascularization. Based on our results, we propose that “photoreceptors at risk” along the margins of EZ loss (and before the development of structural abnormalities) may be the initial source of signaling to the overlying vasculature. We propose that subclinical dysfunction in these photoreceptors may suppress the normal antiangiogenic signaling. As the neurodegenerative process progresses, areas of photoreceptor loss accrue. As EZ loss and antiangiogenic signaling is further disrupted, telangiectasia worsens culminating in the development of outer retinal neovascularization. This is supported by our results that show stepwise increase in overlap between telangiectasia and EZ with advancing disease stage on OCTA, suggesting progressive disruption of these vascular barriers as photoreceptor compromise advances. To further confirm this result, we examined the degree of overlap in our dataset against the recently proposed OCT-based MacTel clinical classification.[16] As shown in **S3 Appendix**, we found that the overlap progressively increased with advancing stage using this scale as well, an encouraging finding that confirms our hypothesis. This result needs to be further validated in larger datasets.

Current models suggest that photoreceptor loss induces vascular change, so it may seem paradoxical that 6 subjects were identified with telangiectasia without evidence of EZ loss. To explain these results, we would like to distinguish photoreceptor loss (marked by EZ loss on OCT) and “photoreceptors at risk” where preclinical dysfunction is not yet visible as structural EZ loss. We propose that these photoreceptors at risk may be responsible for the zones of telangiectasia. The implication of this finding is that DCP telangiectasia could predict the locations of future EZ deficits along its margins. Taken further, the area of telangiectasia at the DCP may therefore be a quantifiable marker of disease severity. Telangiectasia may be particularly relevant as a marker of early stages (I-II) of MacTel, when other established pathologies, such as presence of outer retinal neovascularization, EZ loss, or HF, may not yet be apparent. Several other findings in this study suggest that telangiectasia area may be a useful marker of early disease progression. First, we show that the total area of telangiectasia correlated significantly with visual acuity. Secondly, the qualitative OCTA findings in our 6-month return MacTel subject **(Fig 4)** show that areas of telangiectasia, as well as EZ loss are highly dynamic, and while EZ loss completely disappeared, telangiectasia did not. Future studies are needed to further evaluate whether these dynamic changes reflect improvement or progression of disease.

We also document an association between HF and neovascularization that warrants further discussion. We found that the vast majority of HF were associated with neovascular lesions (either intraretinal or subretinal). The presence of HF could signal underlying neovascularization, which, especially if intraretinal, may go unnoticed when OCTA is not available. This finding is supported by a recent OCTA-based study using the presence of HF as an identifying feature of neovascularization in MacTel.[20] In line with our findings, this study also found a correlation between progressive vascular abnormalities (culminating with neovascularization as marked by HF) and progressive EZ loss.

Our study should be considered with an understanding of its limitations. Our sample size in a single center (38 eyes) is relatively small, consistent with the low prevalence of MacTel. We used a manual method to outline and quantify areas of telangiectasia in this study, which had sufficient inter-rater agreement (ICC = 0.85), but could still benefit from automated methods to improve accuracy and reliability. OCTA stages, as used in this study, have not been widely validated and their correspondence with disease severity remain unknown–another valuable area of future study. For this reason, we also examined our findings using the recently proposed OCT-based clinical classification from Chew et al.[16] Moreover, our cross-sectional study was designed to evaluate disease at a single point in time, so we are not able to evaluate how EZ loss and telangiectasia evolve over time in an individual. Future studies with serial multimodal imaging are needed to further explore these neurovascular pathologic relationships over time. Moreover, future investigation with AO-SLO for subtle photoreceptor changes at the margins of EZ loss would be valuable to investigate the hypothesis and anatomic correlates of “photoreceptors at risk” in MacTel.

In conclusion, this study offers new insight into the early pathogenesis of MacTel and fits well within the current understanding of disease pathogenesis. We found an evolving topographical relationship between EZ loss and telangiectasia, which increased as a function of disease progression from minimal overlap in nonproliferative to complete overlap in proliferative stages, where HF are a key feature. Furthermore, we found the degree of overlap progressively increases with increasing stage based on the OCTA-based classification as well as the recently proposed OCT classification.[16] Finally, we found that the total area of DCP telangiectasia and its overlap with EZ loss correlate inversely to visual acuity and that telangiectasia may present prior to EZ loss in MacTel. Overall, we propose that DCP telangiectasia and the degree of overlap with EZ loss may present two novel parameters that could provide important insights in the early stages of MacTel and suggest future longitudinal studies to examine this relationship, as well as the concept of “photoreceptors at risk” in greater detail.

## Supporting information

S1 Appendix Abstract for Chew et al. classification. (DOCX)

S2 Appendix Staging of MacTel using OCT-based Chew et al. criteria. (DOCX)

S3 Appendix Degree of overlap across disease stage using OCT-based Chew et al. criteria. Mean overlap increases with increasing stage.
(DOCX)

## References

[1] Powner MB, Gillies MC, Zhu M, Vevis K, Hunyor AP, Fruttiger M. Loss of Muller's cells and photoreceptors in macular telangiectasia type 2. Ophthalmology. 2013;120(11):2344–52. Epub 2013/06/19. 10.1016/j.ophtha.2013.04.013 . 23769334

[2] Charbel Issa P, Gillies MC, Chew EY, Bird AC, Heeren TF, Peto T, et al Macular telangiectasia type 2. Progress in retinal and eye research. 2013;34:49–77. Epub 2012/12/12. 10.1016/j.preteyeres.2012.11.002 23219692 PMC3638089

[3] Spaide RF, Klancnik JM Jr., Cooney MJ. Retinal vascular layers in macular telangiectasia type 2 imaged by optical coherence tomographic angiography. JAMA ophthalmology. 2015;133(1):66–73. Epub 2014/10/16. 10.1001/jamaophthalmol.2014.3950 . 25317692

[4] Gass JD, Oyakawa RT. Idiopathic Juxtafoveolar Retinal Telangiectasis. Archives of Ophthalmology. 1982;100(5):769–80. 10.1001/archopht.1982.01030030773010 7082207

[5] Gass JD, Blodi BA. Idiopathic juxtafoveolar retinal telangiectasis. Update of classification and follow-up study. Ophthalmology. 1993;100(10):1536–46. Epub 1993/10/01. . 8414413

[6] Helb HM, Charbel Issa P, RL VDV, Berendschot TT, Scholl HP, Holz FG. Abnormal macular pigment distribution in type 2 idiopathic macular telangiectasia. Retina (Philadelphia, Pa). 2008;28(6):808–16. Epub 2008/06/10. 10.1097/IAE.0b013e31816d81aa . 18536596

[7] Spaide RF, Klancnik JM Jr, Cooney MJ. Retinal vascular layers imaged by fluorescein angiography and optical coherence tomography angiography. JAMA ophthalmology. 2015;133(1):45–50. Epub 2014/10/16. 10.1001/jamaophthalmol.2014.3616 . 25317632

[8] Dogan B, Erol MK, Akidan M, Suren E, Akar Y. Retinal vascular density evaluated by optical coherence tomography angiography in macular telangiectasia type 2. International ophthalmology. 2019 Epub 2019/01/05. 10.1007/s10792-018-01060-x . 30607862

[9] Chidambara L, Gadde SG, Yadav NK, Jayadev C, Bhanushali D, Appaji AM, et al Characteristics and quantification of vascular changes in macular telangiectasia type 2 on optical coherence tomography angiography. The British journal of ophthalmology. 2016;100(11):1482–8. Epub 2016/01/30. 10.1136/bjophthalmol-2015-307941 . 26823394

[10] Nalci H, Sermet F, Demirel S, Ozmert E. Optic Coherence Angiography Findings in Type-2 Macular Telangiectasia. Turkish journal of ophthalmology. 2017;47(5):279–84. Epub 2017/11/08. 10.4274/tjo.68335 29109897 PMC5661178

[11] Yannuzzi LA, Bardal AM, Freund KB, Chen KJ, Eandi CM, Blodi B. Idiopathic macular telangiectasia. Archives of ophthalmology (Chicago, Ill: 1960). 2006;124(4):450–60. Epub 2006/04/12. 10.1001/archopht.124.4.450 . 16606869

[12] Toto L, Di Antonio L, Mastropasqua R, Mattei PA, Carpineto P, Borrelli E, et al Multimodal Imaging of Macular Telangiectasia Type 2: Focus on Vascular Changes Using Optical Coherence Tomography Angiography. Investigative ophthalmology & visual science. 2016;57(9):Oct268–76. Epub 2016/07/15. 10.1167/iovs.15-18872 . 27409482

[13] Gaudric A, Krivosic V, Tadayoni R. OUTER RETINA CAPILLARY INVASION AND ELLIPSOID ZONE LOSS IN MACULAR TELANGIECTASIA TYPE 2 IMAGED BY OPTICAL COHERENCE TOMOGRAPHY ANGIOGRAPHY. Retina (Philadelphia, Pa). 2015;35(11):2300–6. Epub 2015/10/07. 10.1097/iae.0000000000000799 . 26441270

[14] Tan GS, Kuehlewein L, Sadda SR, Sarraf D, Schwartz SD. SUBRETINAL NEOVASCULARIZATION IN MACULAR TELANGIECTASIA TYPE 2: OPTICAL COHERENCE TOMOGRAPHIC ANGIOGRAPHY AND TREATMENT RESPONSE. Retinal cases & brief reports. 2015;9(4):286–9. Epub 2015/08/20. 10.1097/icb.0000000000000191 . 26288110

[15] Spaide RF, Klancnik JM Jr., Cooney MJ, Yannuzzi LA, Balaratnasingam C, Dansingani KK, et al Volume-Rendering Optical Coherence Tomography Angiography of Macular Telangiectasia Type 2. Ophthalmology. 2015;122(11):2261–9. Epub 2015/09/01. 10.1016/j.ophtha.2015.07.025 . 26315043

[16] Chew EY, Peto T, Clemons TE, Pauleikoff D, Sallo F, Heeren T, et al Macular Telangiectasia Type 2: A Classification System Using Multi-Modal Imaging. IOVS. 2019;60. ARVO Abstract 1335.

[17] Shen W, Fruttiger M, Zhu L, Chung SH, Barnett NL, Kirk JK, et al Conditional Mullercell ablation causes independent neuronal and vascular pathologies in a novel transgenic model. The Journal of neuroscience: the official journal of the Society for Neuroscience. 2012;32(45):15715–27. Epub 2012/11/09. 10.1523/jneurosci.2841-12.2012 23136411 PMC4014009

[18] Sallo FB, Peto T, Egan C, Wolf-Schnurrbusch UE, Clemons TE, Gillies MC, et al "En face" OCT imaging of the IS/OS junction line in type 2 idiopathic macular telangiectasia. Investigative ophthalmology & visual science. 2012;53(10):6145–52. Epub 2012/08/18. 10.1167/iovs.12-10580 22899757 PMC4608676

[19] Sallo FB, Peto T, Egan C, Wolf-Schnurrbusch UE, Clemons TE, Gillies MC, et al The IS/OS junction layer in the natural history of type 2 idiopathic macular telangiectasia. Investigative ophthalmology & visual science. 2012;53(12):7889–95. Epub 2012/10/25. 10.1167/iovs.12-10765 23092925 PMC4606792

[20] Pauleikhoff D, Gunnemann F, Book M, Rothaus K. Progression of vascular changes in macular telangiectasia type 2: comparison between SD-OCT and OCT angiography. Graefe's archive for clinical and experimental ophthalmology = Albrecht von Graefes Archiv fur klinische und experimentelle Ophthalmologie. 2019 Epub 2019/05/17. 10.1007/s00417-019-04323-0 . 31093765

[21] Runkle AP, Kaiser PK, Srivastava SK, Schachat AP, Reese JL, Ehlers JP. OCT Angiography and Ellipsoid Zone Mapping of Macular Telangiectasia Type 2 From the AVATAR Study. Investigative ophthalmology & visual science. 2017;58(9):3683–9. Epub 2017/07/21. 10.1167/iovs.16-20976 28727884 PMC5518977

[22] Peto T, Heeren TFC, Clemons TE, Sallo FB, Leung I, Chew EY, et al CORRELATION OF CLINICAL AND STRUCTURAL PROGRESSION WITH VISUAL ACUITY LOSS IN MACULAR TELANGIECTASIA TYPE 2: MacTel Project Report No. 6-The MacTel Research Group. Retina (Philadelphia, Pa). 2018;38 Suppl 1:S8–s13. Epub 2017/05/16. 10.1097/iae.0000000000001697 . 28505012 PMC8326288

[23] Rhee KD, Nusinowitz S, Chao K, Yu F, Bok D, Yang X-J. CNTF-mediated protection of photoreceptors requires initial activation of the cytokine receptor gp130 in Müller glial cells. Proceedings of the National Academy of Sciences. 2013;110(47):E4520–E9. 10.1073/pnas.1303604110 24191003 PMC3839707

[24] Chew EY, Clemons TE, Jaffe GJ, Johnson CA, Farsiu S, Lad EM, et al Effect of Ciliary Neurotrophic Factor on Retinal Neurodegeneration in Patients with Macular Telangiectasia Type 2: A Randomized Clinical Trial. Ophthalmology. 2019;126(4):540–9. Epub 2018/10/08. 10.1016/j.ophtha.2018.09.041 . 30292541 PMC8365464

[25] Hu W, Jiang A, Liang J, Meng H, Chang B, Gao H, et al Expression of VLDLR in the retina and evolution of subretinal neovascularization in the knockout mouse model's retinal angiomatous proliferation. Investigative ophthalmology & visual science. 2008;49(1):407–15. Epub 2008/01/04. 10.1167/iovs.07-0870 . 18172119

[26] Hasegawa E, Sweigard H, Husain D, Olivares AM, Chang B, Smith KE, et al Characterization of a spontaneous retinal neovascular mouse model. PLoS One. 2014;9(9):e106507 Epub 2014/09/05. 10.1371/journal.pone.0106507 25188381 PMC4154693

[27] Zhao M, Andrieu-Soler C, Kowalczuk L, Paz Cortes M, Berdugo M, Dernigoghossian M, et al A new CRB1 rat mutation links Muller glial cells to retinal telangiectasia. The Journal of neuroscience: the official journal of the Society for Neuroscience. 2015;35(15):6093–106. Epub 2015/04/17. 10.1523/jneurosci.3412-14.2015 25878282 PMC4397606

[28] Luo L, Uehara H, Zhang X, Das SK, Olsen T, Holt D, et al Photoreceptor avascular privilege is shielded by soluble VEGF receptor-1. eLife. 2013;2:e00324 Epub 2013/06/26. 10.7554/eLife.00324 23795287 PMC3687373

