# Supplementary material for: Overlap between telangiectasia and photoreceptor loss increases with progression of macular telangiectasia type 2
Source: PLoS One. 2019 Oct 28;14(10):e0224393. doi: 10.1371/journal.pone.0224393 (PMC6816569; doi:10.1371/journal.pone.0224393)
Supplement: S1 Appendix — (DOCX) [file pone.0224393.s001.docx]

**S1 Appendix.** Chew et al. Abstract

Title: A New Classification for Macular Telangiectasia type 2 based on multi-modal imaging.

Authors: Emily Y Chew, Tunde Peto, Traci E Clemons, Daniel Pauleikhoff, Ferenc B Sallo, Tjebo Heeren, Catherine A Egan, Peter Charbel Issa, Konstatinos Balaskas, Alan C Bird.

Purpose: To develop and validate a severity scale for the progression of macular telangiectasia (Mac Tel) type 2, a bilateral neurodegenerative disease, using data from multi-modal imaging

Methods: An international, prospective, observational study of the natural history of Mac Tel type 2 (2005-2018) collected best-corrected visual acuity scores and color fundus photographs, fluorescein angiography, fundus autofluorescence images, and optical coherence tomography (OCT) scans for identifying specific features of Mac Tel type 2 at baseline and annual study visits. Images were graded centrally in London, UK. The Decision Tree Method (DTM) was used to analyze multiple covariates to develop a prediction algorithm for the outcome of visual acuity loss in this study. The right eye data were used as the training set, while the left eye data were used for validation. Analyses were conducted initially using the visual acuity score at enrollment and separate analyses were conducted for the visual acuity scores at the last study visit. Validation of the scale was done by analyzing longitudinal data using mixed effects and generalized linear models (GLM) to assess degree of visual acuity loss and progression along the scale such as 1-, 2-step changes or progression to the more severe stage.

Results: We analyzed the imaging data from 1287 and 1300 right and left eyes, respectively with baseline visual acuities scores and again for the last study visual acuity scores. Based on these analyses, morphological characteristics that appeared to have statistically significant impact on visual acuity loss included: presence and location (central or non-central) of both the ellipsoid zone break on OCT and pigment; and the presence of hyper-reflectivity on OCT. Further analyses using the DTM, these 3 factors were placed in combination to create a scale of 7 steps with increasing severity that was again based upon the target of decreasing visual acuity (Table). Using the data on 875 eyes that had longitudinal data, the model results showed good prediction of 5-year progression rates to decreased visual acuity and to more severe stage.

Conclusions: This current revised system provides a classification that considers the structural changes demonstrated with multi-modal imaging with good correlation with visual acuity. Further studies are warranted to study its validity and to assess OCT hyper-reflectivity lesions.

This abstract was presented at the 2019 ARVO Annual Meeting, held in Vancouver, Canada, April 28 - May 2, 2019. Volume 60, Issue 9.
