# Supplementary material for: Overlap between telangiectasia and photoreceptor loss increases with progression of macular telangiectasia type 2
Source: PLoS One. 2019 Oct 28;14(10):e0224393. doi: 10.1371/journal.pone.0224393 (PMC6816569; doi:10.1371/journal.pone.0224393)
Supplement: S2 Appendix — (DOCX) [file pone.0224393.s002.docx]

| **S2 Appendix.** Staging of MacTel using OCT-based Chew et al. criteria | |
| --- | --- |
| Characteristic | Number of eyes |
| Eyes | 38 (20 subjects) |
| Chew et al. Stage  0 |  |
|  | 6 |
| 1 | 3 |
| 2 | 3 |
| 3 | 3 |
| 4 | 8 |
| 5 | 0 |
| 6 | 15 |
| Stage 0: no EZ loss, no pigment, no HF  Stage 1: noncentral EZ loss, no pigment, no HF  Stage 2: central EZ loss, no pigment, no HF  Stage 3: central EZ loss, noncentral pigment, no HF  Stage 4: central or noncentral EZ loss, HF  Stage 5: central pigment, no neovascularization  Stage 6: central pigment, neovascularization | |
