# Supplementary material for: Overlap between telangiectasia and photoreceptor loss increases with progression of macular telangiectasia type 2
Source: PLoS One. 2019 Oct 28;14(10):e0224393. doi: 10.1371/journal.pone.0224393 (PMC6816569; doi:10.1371/journal.pone.0224393)
Supplement: S3 Appendix — Mean overlap increases with increasing stage. (DOCX) [file pone.0224393.s003.docx]

**
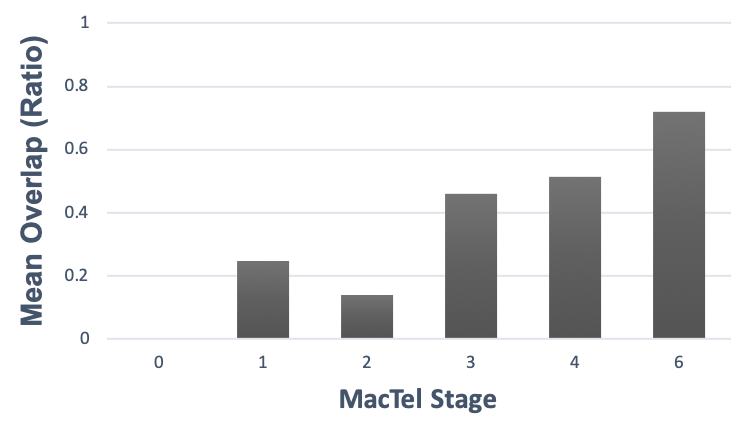
S2 Appendix.** Degree of overlap across disease stage using OCT-based Chew et. al criteria; mean overlap increases with increasing stage.
